# Profile of the main bioactive compounds and *in vitro* biological activity of different solvent extracts from *Ginkgo biloba* exocarp

**DOI:** 10.1039/d0ra09490k

**Published:** 2020-12-21

**Authors:** Na Cui, Liangliang Zhang, Meiping Quan, Jianguo Xu

**Affiliations:** School of Food Science, Shanxi Normal University Linfen 041004 China xjg71@163.com; College of Environment and Life Science, Weinan Normal University Weinan 714000 China

## Abstract

In order to make good use of *Ginkgo biloba* exocarps as agricultural residues, the present work was conducted aiming to evaluate the main bioactive compounds and *in vitro* biological activities of different solvent (petroleum ether, ethyl acetate, *n*-hexane, acetone, ethanol, and methanol) *Ginkgo biloba* exocarp extracts. The methanol extracts with the highest content of total phenolics and total flavonoids showed the strongest antioxidant and antibacterial activities. *n*-Hexane extracts had the lowest total phenolics, flavonoids and antioxidant activities, however, it exhibited moderately high antibacterial activities compared to other extracts. More interestingly, the *n*-hexane extracts with the highest ginkgolic acid content had the strongest inhibitory ability on HepG2 cell viability, and then ethyl acetate, petroleum ether, acetone, ethanol, and methanol extracts. The results showed that bioactive compounds and biological activities of extracts from *Ginkgo biloba* exocarp were greatly affected by the extraction solvents. Therefore, the selective extraction from *Ginkgo biloba* exocarp is very important for processing and comprehensive utilization of *Ginkgo biloba* exocarp.

## Abstract 2

The extraction solvents had significant effects on total phenolics and flavonoids content in extracts from *Ginkgo biloba* exocarp.

## Graphical abstract

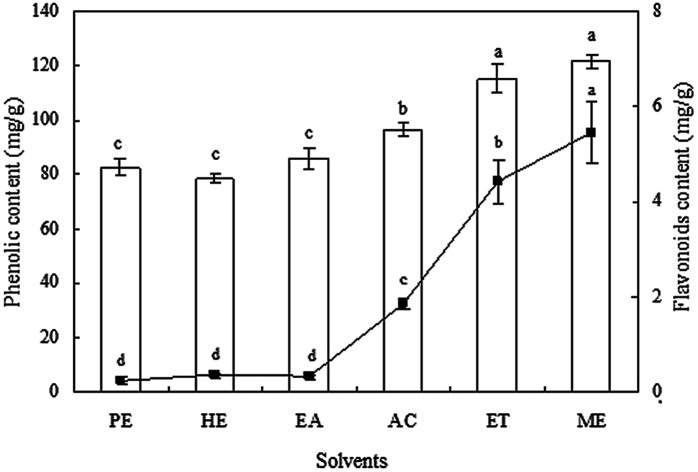

## 1. Introduction

*Ginkgo biloba* L. (Family: Ginkgoaceae) is one of the medicinal plants in the world, which is regarded as a “living fossil” and has existed on the Earth for 200 million years.^1^ As a traditional Chinese herbal medicine, *Ginkgo biloba* L. has high physiological activity in therapies for diseases,^2^ and has a certain ornamental value, so it has been planted in China, Japan, South Korea, France, Germany and other countries.^1,2^ As the main medicinal parts of *Ginkgo biloba*, its leaves have been widely studied. Chemically, the main active components of the *G. biloba* leaves were flavonoids and terpenoids,^3–5^ and the extracts displayed several pharmacological activities including antioxidants, anti-inflammatory, antiallergic, antimicrobial, and cytotoxic antitumour activities.^6–11^

In China, most of *Ginkgo biloba* exocarps are discarded as agricultural residues due to the lack of suitable processing methods after seeds are collected, which not only causes huge waste but also pollutes the environment. Therefore, the reasonable development and utilization of *Ginkgo biloba* exocarp resources can not only produce economic interests but also good social and environmental benefits. Some studies reported that *G. biloba* exocarps had bioactive substances including phenolics, flavonoids, ginkgolic acids and active polysaccharide.^12,13^ In particular, ginkgolic acids were proved to exist in exocarps, leaves, seeds of *Ginkgo biloba*, and its content in the exocarps was higher than that in leaves, seeds of *Ginkgo biloba*.^14^ The main components of ginkgolic acids are five different 6-alkyl salicylic acids, whose alkyl substituents are C13:0, C15:0, C15:1, C17:1, and C17:2, respectively.^15^ Besides, there were several reports about the therapeutic effect and biological activities of the extracts from *G. biloba* exocarps including antioxidant,^16^ antibacterial,^12^ antitumor activity,^17,18^ larvicidal activity,^19^ immunomodulatory,^13^ anti-inflammatory and oestrogenic activities, as well as vascular activities.^20–22^

The phytochemical compositions occur in different concentrations and proportions in plant extracts because of the different extraction solvents and processes, which resulted in the differences in biological activities of extracts.^5,23–25^ At present, there are few reports about the effects of different extraction solvents on phytochemical components and activities of plant exocarp extracts. Therefore, this study was to investigate relationship between different extraction solvents and total content, biological activities *in vitro* of the phenolics, flavonoids, and ginkgolic acids in *Ginkgo biloba* exocarp, which would provide some foundational information for the developing and application of *Ginkgo biloba* exocarps in the food industry.

## 2. Materials and methods

### 2.1 Plant materials and reagents

The exocarp of *Ginkgo biloba* was collected from Linfen City, Shanxi Province, China in October, 2017. They were air-dried at 50 °C in a drying oven, ground into fine powder by a micro plant grinding machine (FZ102; Tianjin Taisite Instruments, Tianjin, China), and stored at −20 °C until use. The gallic acid, Aflatoxin B1 (AFB1), MTT Assay Kit, rutin standard and 2,4,6-tri-(2-pyridyl)-*s*-triazine (TPTZ) were from Sigma (United States). The total ginkgolic acid standard (C13:0, 11.5%; C15:1, 45%; C17:2, 2%; C15:0, 3%; C17:1, 38.5%) (purity > 99%) was purchased from Schwabe Co., Ltd. (Germany).

### 2.2 Microbial strains and culture

Four strains including *Staphylococcus aureus* ATCC 25923, *Bacillus subtilis* ATCC 6051, *Escherichia coli* ATCC 25922 and *Salmonella typhimurium* ATCC 19430 were used for the study. They were obtained from the college of Life Science, Shanxi Normal University. Each strain was cultured in nutrient agar (NA) and nutrient broth (NB) mediums at 37 °C.

### 2.3 Cell cultures

HepG2 cell line can maintain several liver metabolic functions, and is considered an appropriate model for hepatotoxicity studies.^26^ HepG2 cells were purchased from Shanghai cell bank, Chinese Academy of Sciences. These cells were maintained as sub-confluent monolayers in DMEM (Gibco, USA) with 2 mM glutamine, 10% fetal bovine serum (Gibco, USA), 1% non-essential amino acids and 1% penicillin-streptomycin.

### 2.4 Preparation of extracts

Extracts were extracted using solvents of different polarities including petroleum ether, ethyl acetate, *n*-hexane, acetone, ethanol, and methanol. Fifty-gram samples were ground, and then blended with 500 mL solvents. The mixture was shaken with a laboratory rotary shaker (JB50-D, Shanghai Shengke Instruments, Shanghai, China) for 4 h (120 rpm, 20 °C), and then centrifuged for 10 min (6000*g*, 4 °C) in a centrifuge (Eppendorf 5417R, Germany). After centrifugation, the supernatants were collected, and dried at 30 °C by vacuum freezing dryer (SCIENTZ-30ND, Ningbo Xinzhi Biotechnology Co. LTD, Ningbo, China). All extracts were preserved in the refrigerator at −4 °C.

### 2.5 Determination of content of total phenolic and flavonoid

The content of total phenolic and flavonoid was determined according to the methods as described by Feng and Xu.^25^ Standard curve was calibrated by gallic acid and rutin respectively.

### 2.6 HPLC analysis

The analyses were performed on a Waters 1525 HPLC system (Waters, Milford, MA) equipped with Waters 2487 Diode Array Detector (DAD). The reversed-phase C_18_ column (Alltech, Allsphere ODS-2, 5 μm, 250 mm × 4.6 mm) was used to separate at 30 °C. The mobile phase was 2% acetic acid in water–methanol (10 : 90). The detection wavelength was set at 310 nm. The 20 μL extracts were analyzed by injection at a constant flow rate of 1.0 mL min^−1^. The retention time, UV-vis and mass spectra of ginkgolic acids were compared with standard substance to identify and quantify the acid in the samples.

### 2.7 Determination of antioxidant activity

#### 2.7.1 DPPH assay

The scavenging activity of sample on DPPH radicals was evaluated according to the method of Xu, Hu, and Liu.^27^ Put simply, the 0.5 mL samples of different concentrations were added to 2.5 mL 60 μM DPPH and incubated for 30 min at room temperature. Following incubation, the absorbance was read at 517 nm. The scavenging activity of the sample on DPPH radicals was expressed by EC_50_ value that was the concentration of extract that made DPPH radicals to reduce 50%.

#### 2.7.2 ABTS assay

The scavenging activity of sample on ABTS cation radical was determined according to the method reported by Xu *et al.*^27^ Briefly, ABTS radicals were generated by the reaction of 7 mM ABTS in H_2_O with 2.45 mM potassium persulfate for 24 h in the dark at room temperature. The ABTS radicals reagent was then diluted with methanol to 0.700 ± 0.050 absorbance at 732 nm. The 100 μL sample of different concentration was added to 2.0 mL diluted ABTS^+^ solution. After 6 min, the absorbance was read at 732 nm against a blank. The radical scavenging rate and EC_50_ value of sample were calculated as DPPH assay.

#### 2.7.3 Ferric reducing antioxidant power (FRAP) assay

The reducing ability was determined according to the method described by Feng and Xu.^25^ The FRAP reagent was prepared from 300 mM pH 3.6 sodium acetate buffer, 10 mM TPTZ solution in 40 mM HCl and 20 mM FeCl_3_ solution in proportions of 10 : 1 : 1 (v/v), respectively. Then 0.1 mL of the tested sample solution was mixed with 1.8 mL of FRAP reagent and 3.1 mL ultra-pure water. After incubation for 30 min at 37 °C, the absorption of the mixture was measured at 593 nm. The standard curve was constructed using FeSO_4_ solution, and FRAP value was expressed as micromoles Fe(ii) per gram DW.

### 2.8 Determination of antibacterial activity

#### 2.8.1 Agar disc diffusion assay

The antibacterial activity was determined by the method described by Diao *et al.*^28^ with some modifications. Briefly, 100 μL bacterial suspension with a concentration of 10^7^ CFU per mL was taken and uniformly smeared on NA medium by a sterile coating stick. The Whatman No. 1 sterile filter paper discs (6 mm diameter) were placed on the inoculated plates, and then 100 μL of the sample was loaded on filter paper discs. The diameter of zone of inhibition (ZOI) on the inoculated plates were measured after being cultured at 37 °C for 24 h.

#### 2.8.2 Minimum inhibitory concentration (MIC) assay

The MIC was measured according to the method reported by Diao *et al.*^28^ Firstly, extract samples were filtered and sterilized by 0.22 mm Millipore filters, and then added into sterile tubes containing sterile NB medium to obtain different final concentrations. Then 100 μL of the bacterial suspension (approximately 1 × 10^7^ CFU per mL) was transferred to each incubation tube. After incubation at 37 °C for 24 h, the growth status of microorganism in each tube was checked. The lowest concentration of the extracts that demonstrated no visible growth after 24 h of incubation was selected as its MIC for this study.

### 2.9 MTT reduction assay

The viability of HepG2 cells was measured according to MTT reduction assay described by Grollino *et al.*^11^ with some modifications. The HepG2 cells (1.0 × 10^4^ mL^−1^) were inoculated in 96-well plates for 24 h and then were dealt with exocarp extracts for 48 h. The untreated cells, DMSO (1%) treated cells, and 10 mM or 100 mM AFB1 treated cells (positive control) were processed according to the instructions of manufacturer and used in all experiments. These cells were processed according to the manufacturer's instructions. MTT solution (10 mL, 0.5 mg mL^−1^) was added to each well, and then plates were read fluorescence after incubating for 60 min at 37 °C before. The cell viability (%) was expressed as the mean fluorescent intensity of experimental group/solvent control group. The inhibition ability of the sample on HepG2 cells was expressed by IC_50_ value. IC_50_ value is the effective concentration at which the viability of HepG2 cells is inhibited by 50%.

### 2.10 Statistical and data analysis

All experiments were conducted three times independently and the experimental data were expressed as mean ± standard deviation (SD). One-way analysis of variance (ANOVA) and Duncan's multiple range test were carried out to determine significant differences (*p* < 0.05) between the means by SPSS (version 17.0).

## 3. Results and discussion

### 3.1 Contents of total phenolics and flavonoids

**Fig. 1:**
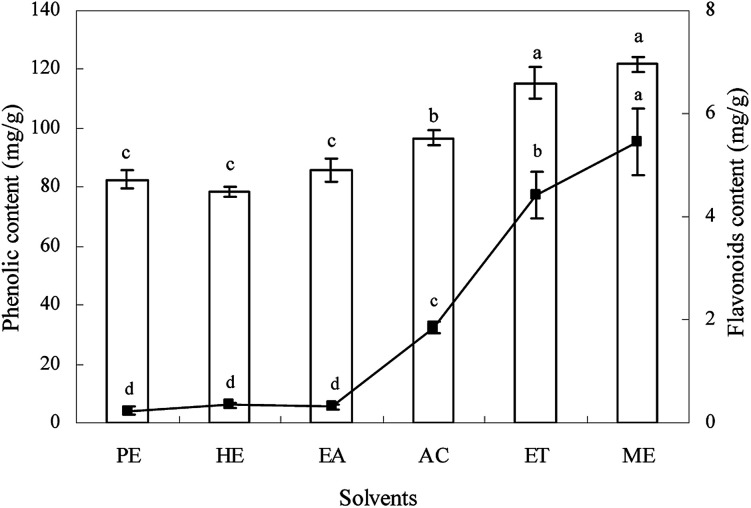
The contents of total phenolics (bar) and flavonoids (line) of different solvent extracts from *Ginkgo biloba* exocarp.

The content of total phenolics and flavonoids in different extracts from *Ginkgo biloba* exocarp are given in Fig. 1. Results showed that extraction solvents had significant effect on total phenolic content and ranged from 78.4 to 121.7 mg g^−1^ (gallic acid equivalent per dried weight). The highest total phenolic content was obtained in methanol and ethanol extracts, followed by acetone, ethyl acetate, petroleum ether, and hexane extracts.

The content of flavonoids was also influenced significantly by solvents (*p* < 0.05) with varying from 0.24 to 5.45 mg g^−1^ (rutin equivalent per dried weight). The methanol extract had the highest flavonoids, followed by ethanol, acetone, hexane, ethyl acetate, and petroleum ether extracts. However, there was no significant difference both in total phenolic and flavonoids content among hexane, ethyl acetate, and petroleum ether extracts. For each extract, the content of total phenolics was much higher than that of flavonoids, indicating that *Ginkgo biloba* exocarp had higher total phenolics, which may be something different to *Ginkgo biloba* leaf that possessed higher total flavonoids.^2,3,5^ In this study, high total polyphenols and flavonoids content were found in methanol extracts, which showed that methanol was the better solvent for extracting phytochemicals from *Ginkgo biloba* exocarp. This was basically similar to some previous reports on other plants.^24,29,30^ These differences in total phenolics and flavonoids content in different extracts from *Ginkgo biloba* exocarp may be attributed to differences in the chemical structure, polarity, dielectric constant of solvents*.*^31^

### 3.2 Contents of ginkgolic acids

**Fig. 2:**
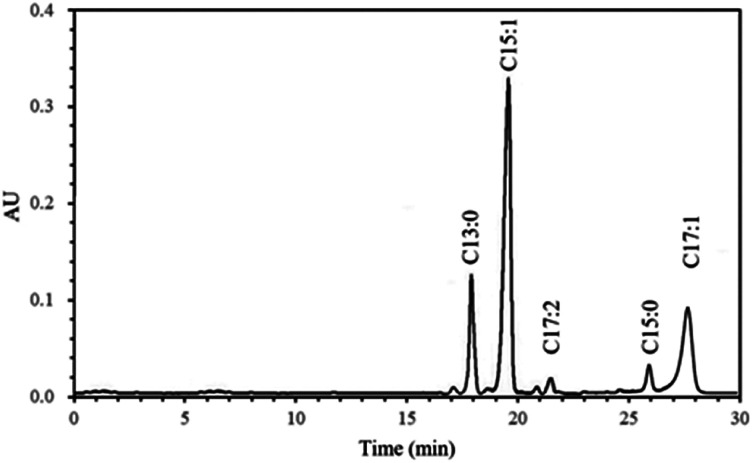
HPLC chromatogram of methanol extract from *Ginkgo biloba* exocarp.

Ginkgolic acid is another important physiological component of *Ginkgo biloba* besides flavonoids and terpene trilactones. HPLC chromatogram of ginkgolic acids in methanol extract is shown in Fig. 2, and the quantitative analytical results of the ginkgolic acids extracted from *Ginkgo biloba* exocarp are shown in Table 1.

**Table 1:** The content (mg per g DW) and relative content (%) of ginkgolic acids of different extracts from *Ginkgo biloba* exocarp^a^

	Ginkgolic acids					
	C13:0	C15:1	C17:2	C15:0	C17:1	Total
Petroleum ether	11.2 ± 0.6a	23.5 ± 1.6ab	1.7 ± 0.2a	2.9 ± 0.6a	8.2 ± 0.3ab	47.5 ± 3.3a
	23.6	49.4	3.6	6.1	17.3	100
*n*-Hexane	11.6 ± 1.0a	26.1 ± 1.3a	1.8 ± 0.3a	3.2 ± 0.5a	9.1 ± 0.4a	51.8 ± 3.5a
	22.4	50.4	3.4	6.2	17.6	100
Ethyl acetate	11.1 ± 0.8ab	24.2 ± 1.4ab	1.6 ± 0.4a	2.4 ± 0.2a	8.5 ± 0.5ab	47.8 ± 3.3a
	23.2	50.6	3.3	5.0	17.9	100
Acetone	9.5 ± 0.2bc	22.3 ± 1.2bc	1.8 ± 0.7a	2.9 ± 0.4a	7.8 ± 0.2b	44.3 ± 2.7 ab
	21.4	50.3	4.1	6.5	17.6	100
Ethanol	8.7 ± 0.4c	19.6 ± 0.5cd	1.4 ± 0.3a	2.4 ± 0.4a	6.5 ± 0.4c	38.6 ± 2.0bc
	22.5	50.8	3.6	6.2	16.8	100
Methanol	8.2 ± 0.3c	18.1 ± 0.8d	1.3 ± 0.5a	2.5 ± 0.2a	6.1 ± 0.3c	36.2 ± 2.1c
	22.7	50.0	3.6	6.9	16.9	100

a Values of the content are represented as mean ± standard deviation of triplicates. Different small letters within a column indicate significant differences at *p* < 0.05 for the content of ginkgolic acids.

The levels of ginkgolic acids C13:0, C15:1, C17:2, C15:0 and C17:1 were also influenced by extraction solvents. The *n*-hexane extract had the highest total ginkgolic acid content, followed by ethyl acetate and petroleum ether extracts, the lowest for ethanol and methanol extracts. This result was supported by previous study that hexane had a selective extractability and the extraction efficiency reached up to 98.5% for ginkgolic acids, which was better than light petroleum and cyclohexane.^15^ Some studies reported that ginkgolic acid C15:1 was the highest, followed by C13:0 and C17:1, which accounted for more than 90% of the total ginkgolic acids.^24,32^ In the present study, the same result was found for each extract, indicating that solvents had no significant effect on composition of ginkgolic acids though affected its content. Therefore, the content of ginkgolic acids C15:1, C13:0 and C17:1 may have a direct effect on the total ginkgolic acid content, which was proven by the present result that the effect of solvents on the content of ginkgolic acids C15:1, C13:0 and C17:1 was all similar to total ginkgolic acids. However, solvents had no significant effect on ginkgolic acids C17:2 and C15:0 from *Ginkgo biloba* exocarp. Besides, the content of ginkgolic acids was also influenced by extraction methods or procedure, analytical methods, the source and origin of raw materials.^5,15,32^

### 3.3 DPPH radical scavenging activity

The scavenging activity of *Ginkgo biloba* exocarp extracts on DPPH was significantly affected by extracting solvents (Table 2). The EC_50_ values of extracts from *Ginkgo biloba* exocarp ranged from 6.3 to 43.5 μg mL^−1^, showing that the scavenging activity of extracts on DPPH radicals were found to be in the following order: methanol > ethanol > acetone > petroleum ether > ethyl acetate > *n*-hexane extracts. However, there was no significant difference in DPPH radical scavenging activity among these extracts.

**Table 2:** The antioxidant activities of different extracts from *Ginkgo biloba* exocarp^a^

	DPPH (EC_50_, μg mL^−1^)	ABTS (EC_50_, μg mL^−1^)	FRAP (μmol Fe(ii) per g)
Petroleum ether	38.6 ± 3.1b	192.3 ± 10.5b	30.6 ± 2.4d
*n*-Hexane	43.5 ± 3.4b	221.5 ± 15.2a	18.4 ± 1.6e
Ethyl acetate	40.8 ± 2.5b	180.5 ± 12.6b	33.6 ± 3.0d
Acetone	10.8 ± 0.6a	63.4 ± 5.2c	61.8 ± 1.5c
Ethanol	6.9 ± 0.4a	33.5 ± 4.4d	82.5 ± 7.4b
Methanol	6.3 ± 0.5a	28.7 ± 3.5d	135.6 ± 5.2a

a Values are represented as mean ± standard deviation of triplicates. Different letters within a column indicate significant differences at *p* < 0.05.

### 3.4 ABTS cation radical scavenging activity

The ABTS scavenging activity of different *Ginkgo biloba* exocarp extracts is presented in Table 2. The EC_50_ values of different extracts on scavenging ABTS ranged from 28.7 to 221.5 μg mL^−1^. Ethanol and methanol extracts showed the highest scavenging activity, with an EC_50_ value of 28.7 μg mL^−1^ and 33.5 μg mL^−1^ respectively, followed by acetone, petroleum ether, ethyl acetate extracts and *n*-hexane extracts. Similar to DPPH assay, the scavenging activity of different extracts against ABTS radical increased dose-dependently at certain concentrations.

### 3.5 Ferric reducing antioxidant power (FRAP)

The ability of *Ginkgo biloba* exocarp extracts to reduce iron(iii) to iron(ii) was determined. It can be observed from Table 2 that different extracts of *Ginkgo biloba* exocarp possessed different reducing capacity, and there was a significant difference (*p* < 0.05) among different extracts*.* In this study, the reducing capacity extracts from *Ginkgo biloba* exocarp ranged from 18.4 to 135.6 μmol g^−1^ (*n*_Fe(ii)_ per dried weight), in which methanol extract had the highest ferric reducing capacity and *n*-hexane extract had the lowest ferric reducing capacity. These results indicated that extracts from *Ginkgo biloba* exocarp had a potency to donate electron to reactive free radicals, terminating the free radical chain reactions.^27^

Generally speaking, bioactive substances including phenolics and flavonoids contributed to the antioxidant activities of extracts. The differences in DPPH, ABTS scavenging activity and FRAP of different *Ginkgo biloba* exocarp extracts may be come from differences in the content of total polyphenols, flavonoids and ginkgolic acids. In addition, each component had own antioxidant activity, and there could be differences in antioxidant activity among different component. As a mixture, the antioxidant activities of extracts were also influenced by their components while different extraction solvents resulted in differences in components of extracts because of differences in the polarity and dielectric constant. These results also indicated complexity of bioactive substances from *Ginkgo biloba* exocarp.

### 3.6 Antibacterial activities

The results of the antibacterial activity of different extracts of *Ginkgo biloba* exocarp are given in Table 3. The ZOI values for *S. aureus*, *B. subtilis*, *S. typhimurium*, and *E. coli* strains, which were sensitive to the different extracts, were in the range of 19.2–22.5 mm, 23.5–27.1 mm, 18.9–21.8 mm and 15.5–21.0 mm, respectively. The MIC values for these tested strains were in the range of 5–40 mg mL^−1^ (Table 3). The higher ZOI value and the lower MIC value were found in the extracts with strong antibacterial activity. On the whole, of these extracts, methanol, ethanol, *n*-hexane extracts exhibited better antibacterial activity against tested strains, followed acetone extract, the lowest for petroleum ether and ethyl acetate extracts.

**Table 3:** The ZOI (mm) and MIC values (mg mL^−1^) of different extracts from *Ginkgo biloba* exocarp^a^

	*S. aureus*		*B. subtilis*		*S. typhimurium*		*E. coli*	
	ZOI	MIC	ZOI	MIC	ZOI	MIC	ZOI	MIC
Petroleum ether	19.3 ± 0.7c	20	23.8 ± 0.8c	10	19.7 ± 0.5b	20	16.2 ± 0.7cd	40
*n*-Hexane	21.3 ± 0.6ab	10	25.6 ± 0.5ab	5	20.2 ± 0.5ab	10	19.6 ± 0.5ab	20
Ethyl acetate	20.1 ± 0.4bc	20	23.5 ± 0.7c	10	19.8 ± 0.6b	40	15.5 ± 1.0d	40
Acetone	19.2 ± 0.5c	20	24.2 ± 0.6bc	5	18.9 ± 1.2b	20	18.2 ± 0.8bc	20
Ethanol	22.5 ± 0.6a	10	26.3 ± 0.4a	5	20.5 ± 0.6ab	20	20.4 ± 0.6ab	20
Methanol	22.2 ± 0.3a	10	27.1 ± 0.5a	5	21.8 ± 0.7a	20	21.0 ± 1.1a	20

a Values of ZOI are represented as mean ± standard deviation of triplicates. Different small letters within a column indicate significant differences at *p* < 0.05.

In this study, all extracts from *Ginkgo biloba* exocarp exhibited greater antibacterial activity on Gram-positive strains compared to Gram-negative strains. One reason may be that Gram-negative bacteria possessed an outer membrane composed of lipopolysaccharide and lipoprotein. The outer membrane was selectively permeable and thus restricted diffusion of hydrophobic compounds access to the underlying structures,^33^ which made the Gram-positive generally susceptible to some plant extracts than the Gram-negative bacteria.^34,35^

### 3.7 Cell viability

**Fig. 3:**
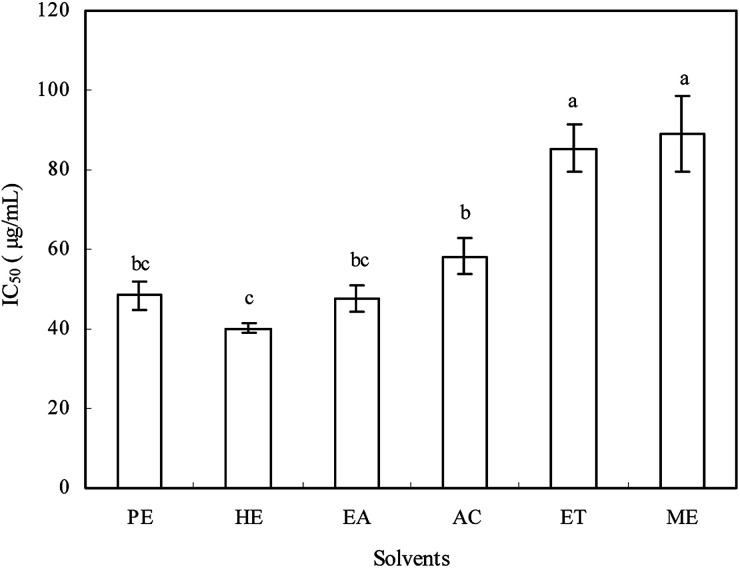
The inhibitory effects of different solvent extracts from *Ginkgo biloba* exocarp on HepG2 cells *in vitro*.

To investigate the potential effect of the extracts from *Ginkgo biloba* exocarp on cell viability, the HepG2 cells were treated with extracts of different concentrations (ranging from 10 to 200 μg mL^−1^) for 48 h. The IC_50_ values of extracts against HepG2 cells ranged from 40.2 to 88.9 μg mL^−1^ (Fig. 3), indicating that the inhibition ability on HepG2 cell viability was found to be in the following order: *n*-hexane > ethyl acetate > petroleum ether > acetone > ethanol > methanol extracts, which was different with their antioxidant activities. In addition, all extracts had inhibitory effect on HepG2 cells in a dose-dependent manner (not shown). Han *et al.*^21^ reported that *Ginkgo biloba* exocarp extract had significant inhibitory effect on Lewis lung cancer cells in a dose-dependent manner and the IC_50_ was 156.25 μg mL^−1^.

### 3.8 Correlation between active compounds and biological activities

To further investigate their interrelationship, the correlation between the active compounds and biological activities was analyzed. As depicted in Table 4, the DPPH, ABTS, FRAP, and antibacterial activity were highly and positively correlated to both total phenolics and flavonoids (*R* ≥ 0.6498), indicating total phenolics and flavonoids contributed to the antioxidant and antibacterial activities of extracts. Similar results also have been reported by other researchers.^23,36^ However, there was a negative correlation between the inhibitory effects on HepG2 cells and the content of phenolics and flavonoids. On the contrary, ginkgolic acids had a significantly correlation (*p* < 0.05) with the changes in the inhibitory effects on HepG2 cells while they were correlated negatively with the antioxidant and antibacterial activities.

**Table 4:** Correlation analysis of the content of polyphenols, flavonoids, ginkgolic acids and bioactivity of different extracts^a^

	Antioxidant activities			Antibacterial activity		Inhibitory effects
	DPPH	ABTS	FRAP	*S. typhimurium*	*B. subtilis*	
Phenolics	0.9859**^b^	0.9944**	0.9590**	0.6498	0.7494	−0.9682**
Flavonoids	0.9841**	0.9979**	0.9572**	0.7181	0.8381*	−0.9381**
Ginkgolic acids	−0.9634**	−0.9722**	−0.9554**	−0.6099	−0.6662	0.9854**

a Values are correlation coefficient *R*.

b Significantly different: **p < 0.01, *p < 0.05.

But it should be noted that the correlation did not verily reflect the contribution of active compounds to biological activities. In fact, ginkgolic acids was a mixture of phenolic acids, so negative correlation of ginkgolic acids with the antioxidant and antibacterial activities didn't mean that they had not the antioxidant and antibacterial activities. As a mixture, the biological activities of extracts was a comprehensive measure, was a result that impacted by content and active ability of all components including other active compounds besides total polyphenols, flavonoids and ginkgolic acids. However, the combinations of different active compounds can result in synergism, additive or antagonistic effects. The study considered effects of total polyphenols, flavonoids and ginkgolic acids on biological activities only from the aspects of their content without active ability of each component and interaction of different active compounds. In addition, presumably there may be other active compounds that had greater activity in extracts. Therefore, the interrelationship and mechanism between these active compounds and biological activities is still needed to be studied further.

## 4. Conclusion

In conclusion, the extracting solvents significantly affected the content of total phenolics, flavonoids, ginkgolic acid and biological activities including antioxidant and antibacterial activities as well as inhibition ability on HepG2 cell viability of *Ginkgo biloba* exocarp. Among these extracts, the methanol extract had the highest total phenolics and flavonoids, and exhibited the strongest antioxidant and antibacterial activities, while hexane extract had the lowest total phenolics and antioxidant activities, and moderately high flavonoids and antibacterial activities compare to other extracts. But interestingly hexane extract was found to have the highest total ginkgolic acid content and the inhibition ability on HepG2 cell viability, followed by ethyl acetate and petroleum ether extracts, the lowest for ethanol and methanol extracts. Therefore, the selective extraction from *Ginkgo biloba* exocarp is very important for obtaining fractions with different bioactivities from *Ginkgo biloba* exocarp. These results provide additional and useful information for exploitation and utilization of *Ginkgo biloba* as well as solving the environmental problem caused by large amount of wasted material.

## Conflicts of interest

There are no conflicts to declare.

## Supplementary Material
